# Assessment of human leukocyte antigen immunogenicity: current methods, challenges and opportunities

**DOI:** 10.1097/MOT.0000000000000544

**Published:** 2018-06-04

**Authors:** Hannah C. Copley, Madhivanan Elango, Vasilis Kosmoliaptsis

**Affiliations:** a Department of Surgery, University of Cambridge and NIHR Cambridge Biomedical Research Centre, Cambridge University Hospitals NHS Foundation Trust, Addenbrooke's Hospital; b NIHR Blood and Transplant Research Unit in Organ Donation and Transplantation at the University of Cambridge, Cambridge, UK

**Keywords:** B-cell epitopes, human leukocyte antigen immunogenicity, humoral alloimmunity, T-cell epitopes

## Abstract

**Purpose of review:**

Donor–recipient human leukocyte antigen (HLA) matching improves outcomes after solid-organ transplantation, but current assessment of HLA incompatibility is inadequate as it does not consider the relative immunogenicity of individual HLA mismatches. In this article, we review existing strategies for assessing HLA immunogenicity and discuss current challenges and future opportunities in this field.

**Recent findings:**

Current HLA immunogenicity algorithms focus primarily on the humoral component of the alloimmune response and aim to determine a measure of ‘dissimilarity’ between donor and recipient HLA. This can be achieved by deriving information from comparison of donor and recipient HLA at the amino acid sequence, structural and/or the physicochemical level, accounting for both B-cell and T-cell pathways of alloreactivity. Substantial evidence now supports the superiority of this molecular definition of HLA incompatibility, over conventional enumeration of HLA antigenic differences, for assessing the risk of humoral alloimmunity and for predicting graft outcomes after transplantation.

**Summary:**

Significant progress has been made in developing computational HLA immunogenicity algorithms that offer exciting opportunities for a more rational approach to determining the degree of donor–recipient HLA incompatibility and to defining HLA-related immunological risk. A number of challenges now need to be overcome to enable their implementation into clinical practice.

## INTRODUCTION

Donor and recipient human leukocyte antigen (HLA) incompatibility is the main immunological barrier to successful organ transplantation. HLA matching has a beneficial effect on solid organ transplant outcomes, including on graft function and on graft and patient survival, although the magnitude of this effect varies in different organs [1]. Due to the extensive polymorphism of the HLA system and the limited size of the donor organ pool, HLA matching is difficult to achieve and most recipients receive HLA mismatched grafts. Moreover, the benefits of minimizing the number of donor–recipient HLA incompatibilities must be balanced against the, often competing, interests of ensuring equity of access to transplantation and of reducing the detrimental effects of prolonging the length of time on the transplant waiting list. Consequently, even though HLA matching is incorporated into many deceased-donor organ allocation schemes, the significance placed on improving HLA compatibility has progressively diminished in favor of increasing reliance on immunosuppression therapies to improve graft outcomes. It is evident, however, that HLA incompatible allografts necessitate the use of heavier immunosuppression, a major cause of recipient morbidity and mortality, and increase the risk of sensitization which can severely limit the opportunity for repeat organ transplantation.

The principles of HLA matching for organ allocation and of evaluating the immunological risk associated with a particular donor–recipient HLA combination have remained largely unchanged for many decades. Histocompatibility assessment, currently focuses almost entirely on HLA-A, HLA-B and HLA-DR loci, is based on counting HLA antigenic differences at the serological level and is predicated on the assumption that all mismatches within an HLA locus are of equal significance to transplant outcomes. However, it is now increasingly being recognized that the conventional approach to assessment of HLA compatibility is inadequate. Central to this notion is the realization that the capacity of a donor HLA to induce immune responses is not an intrinsic property of the mismatched alloantigen but that it is critically dependent on the HLA phenotype of the recipient. Recent advances in molecular sequence technology, increasing availability of high quality, crystallographically resolved, HLA structures and computational approaches to studying B-cell and T-cell epitopes offer exciting opportunities for a more rational approach to determining HLA compatibility and to defining HLA-related immunological risk. In this article, we review existing strategies for assessing HLA immunogenicity, including amino acid sequence, structural and physicochemical approaches, focusing on both B-cell and T-cell pathways of alloreactivity. We also discuss current challenges and opportunities in the field and consider future research directions that might transform clinical practice to maximize the benefits of transplantation. 

**Box 1:**
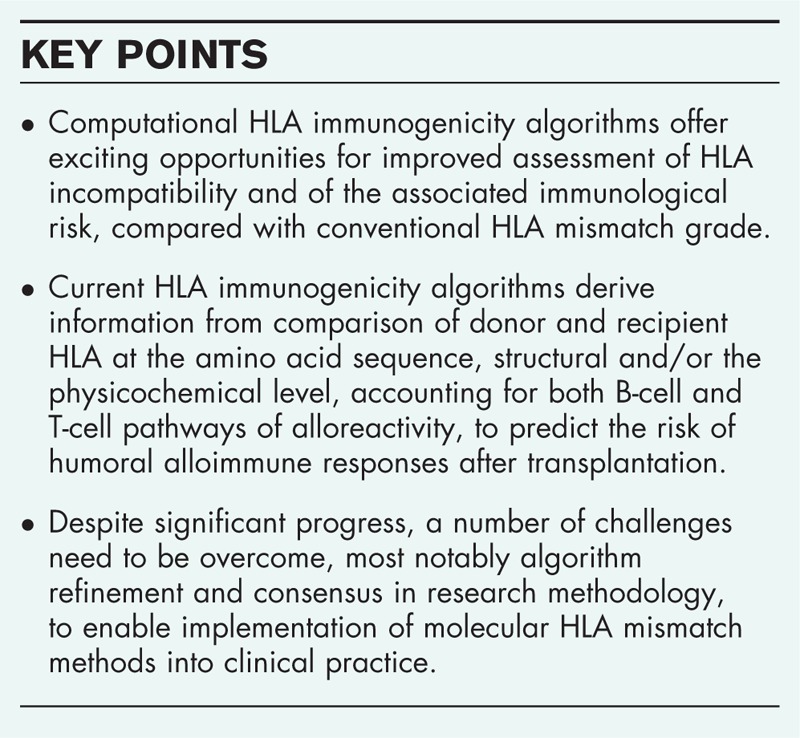
no caption available

## ASSESSMENT OF HUMAN LEUKOCYTE ANTIGEN IMMUNOGENICITY AT THE AMINO ACID SEQUENCE LEVEL

The immunogenicity of nonself-antigenic determinants can be defined by their capacity to induce a host immune response. In the context of transplantation, HLA immunogenicity might be considered at the cellular (T-cell alloreactivity) and/or the humoral (B-cell alloreactivity leading to the development of HLA-specific antibodies) level, although it is evident that these immune responses are interdependent and intrinsically linked. The complexity of T-cell allorecognition and the absence of a readily available and easily interpretable assay to detect allospecific T cells in transplant patients have, so far, hampered the successful development of theoretical algorithms to predict the potential of donor HLA to induce T-cell alloreactivity [2]. In contrast, the detection of alloantibody responses against HLA expressed on donor tissue has driven the field of HLA histocompatibility. The differential capacity of donor HLA to generate alloantibody responses and the favorable graft outcomes in the presence of ‘permissible’ HLA mismatches have long been recognized [3,4], driving attempts to create algorithms that define the potential of donor HLA to induce humoral alloresponses. HLA immunogenicity in the context of humoral alloimmunity is the focus of the present review.

The relationship between amino acid polymorphisms on donor HLA with alloantibody responses and kidney transplant outcomes was recognized over 2 decades ago and these observations have formed the basis of subsequent HLA immunogenicity algorithms [5,6]. Most significant in this regard has been the description of HLAMatchmaker by Duquesnoy *et al.* a computer algorithm that aims to determine the capacity of donor HLA to induce humoral alloimmunity by evaluating differences in the number and location of amino acid polymorphisms at continuous (triplets) and discontinuous (eplets) positions between donor and recipient HLA molecules [7,8]. The fundamental premise of HLAMatchmaker is that eplets, small patches of mismatched amino acids on or near the molecular surface of HLA, are potential immunogenic epitopes that define the specificity of HLA-specific antibodies. To verify this hypothesis, HLAMatchmaker has been used to analyze alloantibody binding profiles in patient sera with a view to describing HLA-specific antibody reactivity based on reactive eplets rather than HLA specificities (reviewed in [9,10]); such eplets, termed ‘antibody-verified’ eplets, are recorded in a publically accessible registry (http://www.epregistry.com.br/). It is evident, however, that this approach is not straight-forward [confounded by the presence of multiple alloantibodies in a patient's serum, variations in single-antigen bead assay analysis – including in the use of mean fluorescence intensity (MFI) cutoff values etc.] and often leads to complex theoretical interpretation, such as the hypothetical requirement for combinations of multiple eplets or of ‘self’ eplets, to explain alloantibody reactivity [11]. It is not, therefore, clear whether B-cell epitopes are adequately defined by HLAMatchmaker eplets and the feasibility of an epitope (eplet)-based approach to donor–recipient matching, and to organ sharing, as has recently been suggested, remains doubtful [12].

The principal hypothesis underpinning theoretical approaches to predicting the risk of development of HLA-specific antibody is that HLA allorecognition by recipient B-cells is more likely the more ‘different’ the donor HLA is compared with recipient HLA molecules. In this regard, HLA immunogenicity algorithms should aim to quantify a measure of ‘dissimilarity’ between donor and recipient HLA. The HLAMatchmaker algorithm has been used successfully in this context as multiple studies have shown an association between the total number of eplets present on HLA class I and II mismatches (termed ‘eplet load’) and the risk of development of HLA-specific antibodies [13^▪^,^14^–^16^] (recently reviewed in [12]). Many studies in this field are often confounded by their retrospective design and the lack of multivariate modeling. Wiebe *et al.*, however, have recently performed a relatively large, single-center, prospective study that benefited from high-resolution HLA typing, information on immunosuppression levels and patient adherence and from serial posttransplant antibody monitoring. Their analysis demonstrated that donor HLA-DR and HLA-DQ eplet load, along with tacrolimus trough levels, were independent predictors of development of donor-specific antibody (DSA) [17^▪▪^]. The clinical utility of this molecular definition of donor–recipient HLA compatibility has been highlighted by studies demonstrating an association between donor HLA eplet load and the development of transplant glomerulopathy, acute kidney graft rejection and graft loss, as well as chronic lung allograft dysfunction and pediatric heart transplant loss [18–21]. It is important to emphasize that the majority of the aforementioned studies on risk of humoral alloimmunity investigated the overall immunogenicity score (eplet load) of mismatches within an HLA locus (the sum of eplets for one or two HLA mismatches within the locus) and the likelihood of a locus-specific alloantibody response (to any of the mismatched alloantigens). The immunological basis of this type of analysis might be inconsistent with the underlying hypothesis in so far as, in cases in which a high eplet load reflects the sum of a low eplet and a high eplet HLA mismatch within the same locus, the alloantibody response might be directed against the low eplet donor HLA. Given that a molecular definition of HLA compatibility requires discrimination between low vs. high immunogenicity single HLA mismatches, future studies should aim to address this potential confounder.

An alternative approach to predicting HLA immunogenicity by assessment of the ‘dissimilarity’ between donor and recipient HLA, based on information derived from amino acid sequence analysis, has also been described. The Cambridge HLA immunogenicity algorithm performs interlocus (for HLA class I) and intralocus (for HLA class II) comparisons between donor and recipient HLA to enumerate all mismatched amino acid sequence polymorphisms on donor HLA which are then scored according to their physicochemical properties (hydrophobicity and electrostatic charge). Analysis of alloantibody responses in highly sensitized patients awaiting kidney transplantation, using this approach, showed that the capacity of donor HLA to induce a humoral response depends not only on the number of its polymorphic residues but also on the physicochemical nature of their side chains [22,23]. In these studies, consideration of only surface accessible amino acid polymorphisms did not improve the power of the model to predict alloantibody responses. Assessment of the electrostatic charge of amino acid polymorphisms on donor HLA-A and HLA-B alloantigens has been shown to correlate independently (accounting for the number of amino acid mismatches) with risk of DSA development in both highly sensitized patients and against HLA class I mismatches expressed on a failed kidney allograft [13^▪^,^23^]. However, due to the correlation between the number of amino acid polymorphisms expressed on a donor HLA and their overall physicochemical disparity, assessment of donor–recipient HLA physicochemical differences at the sequence level has not been shown to provide an advantage, over simple enumeration of amino acid mismatches, in assessing the risk of DSA responses against HLA class II alloantigens [13^▪^,^22^].

**FIGURE 1:**
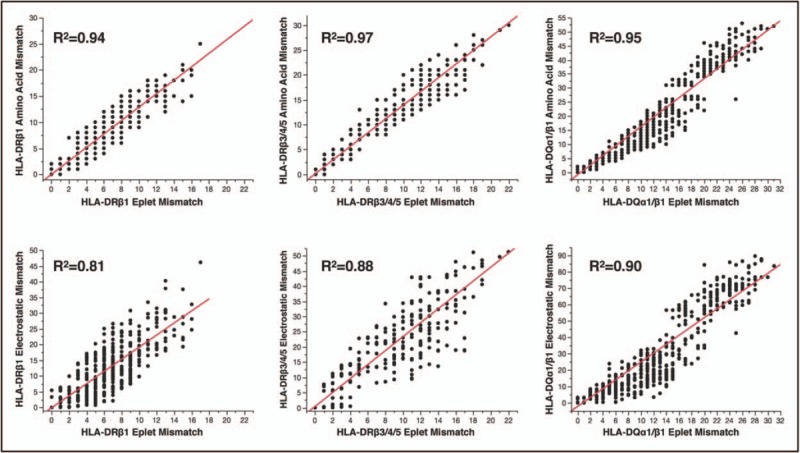
Correlation between amino acid sequence-based human leukocyte antigen mismatch scoring methods; HLAMatchmaker eplet load vs. number of amino acid mismatches or sequence-based electrostatic mismatch score. This figure depicts analyses of the immunogenic potential of human leukocyte antigen class II mismatches in a large cohort of kidney transplant recipients using HLAMatchmaker and the Cambridge human leukocyte antigen immunogenicity algorithm. The strong correlation between number of eplet mismatches (eplet load), amino acid mismatches and sequence-based electrostatic mismatch score for human leukocyte antigen mismatches within the human leukocyte antigen-DRβ_1_, human leukocyte antigen-DRβ_3/4/5_, human leukocyte antigen-DQ_α1β1_ loci is evident. Reproduced with permission [24^▪^].

The aforementioned studies demonstrate that assessment of donor–recipient HLA mismatch at the amino acid sequence level enables quantification of the degree of mismatch improving the precision of immunological risk assessment with DSA development as the immune response readout. The potential benefit of using a particular computational algorithm to assess HLA immunogenicity was recently examined in a cohort of 596 renal transplant patients that were prospectively followed for DSA development against HLA-DR and HLA-DQ mismatches, accounting for immunosuppression therapy and recipient nonadherence [24^▪^]. This study showed that all donor HLA immunogenicity scoring methods, eplet mismatch load or number of amino acid mismatches or sequence-based electrostatic mismatch score (EMS), were significant multivariate correlates of DSA development outperforming conventional HLA mismatch grade for this purpose. No advantage was demonstrated in using one approach over another and each method provided equivalent assessment of immunological risk associated with donor HLA class II mismatches. As might be expected, there was strong correlation between molecular mismatch scores (*R*^2^ = 0.85–0.96) highlighting the fact they all reflect differences in donor–recipient amino acid sequence (Fig. 1).

## ASSESSMENT OF HUMAN LEUKOCYTE ANTIGEN IMMUNOGENICITY AT THE TERTIARY STRUCTURE LEVEL

Even though amino acid sequence-based methods have been successful in predicting the risk of humoral alloimmunity after transplantation and in quantifying donor–recipient HLA incompatibility, sequence information alone provides limited insight into the structural aspects of HLA allorecognition by recipient B-cell receptors. Given the conformational nature of the majority of B-cell epitopes, a fully structural approach might be required to capture and characterize their immunogenic potential. Epitope–paratope interactions are largely governed by short-range electrostatic interactions, such as van der Waals forces, hydrogen bonds and salt-bridges [25], which are important determinants of the affinity and specificity of antibody–antigen binding [26]. It has also been suggested that the process of affinity maturation involves optimization of electrostatic interactions in the B-cell receptor–antigen binding site [26–29]. Previous studies have shown that HLA B-cell epitopes can be described accurately by their unique surface electrostatic potential profiles that help explain serological patterns of HLA-specific antibody binding [30–32]. Given that recipient B-cells do not recognize self HLA molecules, it might be hypothesized that donor HLA with disparate electrostatic potential profiles compared with recipient HLA molecules might be recognized more efficiently by recipient B-cell receptors leading to improved selection and survival of differentiated B-cells during the process of affinity maturation in the germinal center. Indeed, recent insights into the mechanisms that determine the fate decision of proliferating, antigen-activated B-cells at the pregerminal center stage suggested that B-cells with higher affinity to their antigen presented more HLA-peptide to and made longer lasting contact with cognate T follicular helper cells at the B-cell–T-cell border in secondary lymphoid organs, resulting in more T-cell help and differentiation into germinal center B-cells [33,34]. These observations have recently led to the development of a novel HLA immunogenicity algorithm that enables quantitative comparison of surface electrostatic potential properties between donor and recipient HLA at the tertiary structure level (EMS-three–dimensional; a description of the methodology, applied to the study of HLA B-cell epitopes, is provided in [30]). This approach was applied to examine humoral alloimmune responses in healthy females subjected to a standardized injection of donor lymphocytes from their male partner. Preliminary results, published in abstract form [35,36] showed a strong association between the EMS-three-dimensional of donor HLA and donor-specific alloantibody development (Mallon *et al.*[37], article in submission). The algorithm was also used to analyze donor–recipient HLA compatibility in a large cohort of kidney transplant recipients, and a preliminary report suggested a strong association between EMS-three-dimensional and risk of graft failure using a multivariate Cox regression model of graft survival [38]. This is a developing field of research and further work is required to determine the potential of this approach to provide a fully structural description of HLA immunogenicity and whether it might enable improved assessment of donor–recipient histocompatibility compared to conventional HLA matching and to HLA amino acid sequence-based algorithms.

## T-CELL APPROACHES TO PREDICTING HUMAN LEUKOCYTE ANTIGEN IMMUNOGENICITY

**FIGURE 2:**
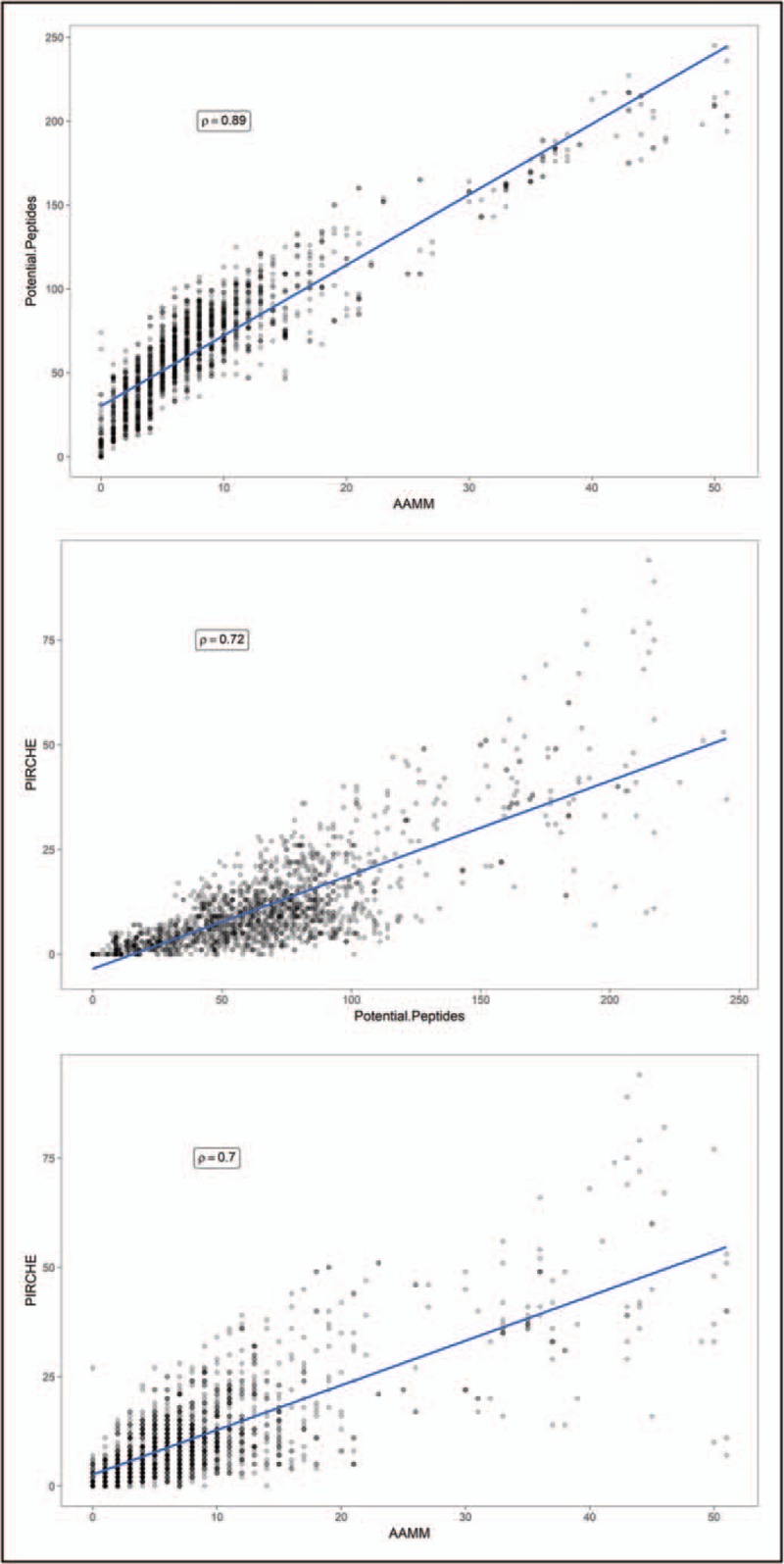
Correlation between predicted indirectly recognizable HLA epitopes and amino acid sequence polymorphism. The figure depicts analysis of HLA (A, B, C, DR, DQ and DP) mismatches in a local cohort of 182 donor–recipient pairs (HLA typed at two-field resolution). HLA mismatches were identified (*n* = 1615) and scored according to the number of possible peptide epitopes (unique, polymorphic nonameric binding cores) that can be derived from donor HLA (irrespective of predicted binding affinity to recipient HLA-DR), the number of predicted indirectly recognizable HLA epitopes (predicted indirectly recognizable HLA epitopes; defined as polymorphic nonameric binding cores with predicted binding to recipient HLA-DR of IC_50_ < 1000 nmol/l), and the number of amino acid mismatches (as defined by the Cambridge HLA immunogenicity algorithm). Donor HLA-derived polymorphic nonameric binding cores were defined as those that differed by at least one amino acid compared with the HLA sequences of the recipient. The NetMHCIIpan version 3.1 algorithm was implemented locally to calculate the predicted indirectly recognizable HLA epitopes scores (the predicted indirectly recognizable HLA epitopes algorithm available via https://www.pirche.org implements a previous version of NetMHCIIpan). For the purposes of this analysis, all HLA mismatches were grouped together. There was high correlation between scores (Spearman's Rho), although this correlation varied for mismatches within individual HLA loci (data not shown). HLA, human leukocyte antigen.

The above-mentioned approaches to determining the immunogenicity of HLA have predominantly focused on the B-cell component of humoral alloimmunity. Proliferation and differentiation of antigen-specific naïve B-cells into memory B-cells and long-lived plasma cells requires T-cell help through linked recognition of antigenic peptides presented in the context of B-cell HLA class II molecules [39]. The implication of this mechanism is that the capacity of recipient HLA class II molecules to present donor-HLA-derived peptides is a critical determinant of the risk of DSA development, and this was supported by observational studies that showed an association between the recipient HLA-DR phenotype and humoral alloresponses to donor HLA class I alloantigens [40,41]. This concept has recently been applied into a computational algorithm that enables enumeration of putative CD4^+^ T-cell epitopes derived from donor HLA (predicted indirectly recognizable HLA epitopes or PIRCHE-II) [42]. PIRCHE implements the NetMHCIIpan program which predicts peptide binding affinity into the groove of an HLA class II molecule based on a neural network method trained on a set of quantitative peptide binding data [43,44]. For a given peptide length, the program identifies potential core nonamer (9-mer) peptides contained within it and outputs the binding affinity of the highest scoring nonamer. In the PIRCHE implementation, predicted nonameric binding cores must be polymorphic compared with the recipient's own HLA molecules. The algorithm enables processing of donor HLA classes I and II alloantigens to derive putative candidate peptide fragments but assessment of peptide binding affinity is currently limited to recipient HLA-DR molecules. Donor-derived candidate peptides with predicted binding of IC_50_ (half maximal inhibitory concentration) less than 1000 nmol/l to recipient HLA-DR are summed to produce the PIRCHE score. The number of PIRCHE peptides derived from HLA class I mismatches was shown to correlate in univariate analyses with development of DSA after graft nephrectomy and after pregnancy, although there was a significant overlap in PIRCHE scores between immunogenic and nonimmunogenic donor HLA [42,45]. In a more recent study that investigated a large cohort of kidney transplant patients (*n* = 2787), the overall PIRCHE score for the total number of donor–recipient HLA-A, HLA-B, HLA-C, HLA-DR and HLA-DQB mismatches (sum of PIRCHE scores derived from every HLA mismatch in a given donor–recipient combination) was an independent predictor of risk of development of DSA (defined as alloantibody against any of the mismatched donor HLA; area-under-the-curve: 0.641), after adjustment for HLAMatchmaker eplet mismatching and for conventional HLA mismatching (there was no data on posttransplant immunosuppression regimens and on patient adherence) [46^▪^]. However, the same independent effect was not confirmed in a similar analysis of a subgroup of these patients (*n* = 1247) with the most complete pretransplant and posttransplant antibody monitoring in which the total number of HLA eplets was the best predictor of risk of DSA responses. It was notable that after analysis of locus-specific alloantibodies, the PIRCHE score did not correlate with risk of development of DSA against individual HLA-A, HLA-B, HLA-C, HLA-DR and HLA-DQB alloantigens when two HLA mismatches were present in the locus and, therefore, further studies are needed to determine whether PIRCHE can be used to predict the immunogenicity of individual HLA mismatches. When the effect of this approach on kidney graft outcomes was analyzed in this study, and more recently in an independent patient cohort [47^▪^], PIRCHE mismatching correlated with graft survival, although it is not clear whether this effect is independent of conventional HLA matching and/or eplet mismatching (only univariate analysis was performed in the Lachmann *et al.*[46^▪^] study and a forward stepwise selection multivariable model – that might not be appropriate in cases of multicollinearity – was used in the Geneugelijk *et al.*[47^▪^] study). The challenge of confirming an independent effect of a novel HLA scoring system on graft survival, over and above that observed with conventional HLA matching, is better appreciated considering the significant correlation between HLA scoring variables (e.g. there was high correlation between PIRCHE scores and HLAMatchmaker scores – Spearman's Rho of 0.75 – and between PIRCHE scores and conventional HLA mismatches in the study by Lachmann *et al.*[46^▪^]). To illustrate the latter observation, we examined the relationship between the number of possible peptide epitopes (unique, polymorphic nonameric binding cores) that can be derived from donor HLA (irrespective of predicted binding affinity to recipient HLA-DR), the number of PIRCHE epitopes (with predicted binding to recipient HLA-DR of IC_50_ <1000 nmol/l) and the number of amino acid polymorphisms present in HLA-A, HLA-B, HLA-C, HLA-DR, HLA-DQ and HLA-DP mismatches within a local cohort of 182 donor–recipient pairs. As shown in Fig. 2, there was high correlation between HLA scoring systems (although there was variation of PIRCHE scores for the same number of amino acid polymorphisms) suggesting that, to an extent, the current PIRCHE approach reflects donor HLA polymorphism at the amino sequence level. Overall, PIRCHE is an interesting approach for assessing potential CD4^+^ T-cell epitopes derived from donor HLA and how this might impact on risk of humoral alloimmunity after transplantation. Further improvements of CD4^+^ T-cell alloreactivity prediction algorithms might arise from consideration of peptide presentation by recipient HLA-DQ and/or HLA-DP and from experimental validation of predicted epitopes that could inform the theoretical design of computational algorithms (e.g. by investigating the predicted peptide binding affinity cutoff values that best reflect experimental observations).

## CONCLUDING REMARKS AND FUTURE DIRECTIONS

There is currently great need to develop and implement clinically applicable algorithms that enable better assessment of donor–recipient HLA incompatibility and of the associated immunological risk, both at a population and at the individual patient level, to maximize the benefits of transplantation. It is evident from the aforementioned studies that significant progress has been made, but there are a number of challenges to overcome to facilitate a sea change in the field. Significant in this regard is the heterogeneity of studies investigating HLA immunogenicity that limits interpretation and synthesis of available evidence. This heterogeneity pertains to a number of factors that introduce uncertainty, including the level of HLA typing resolution (serological, one-field, two-field), Luminex single antigen bead output interpretation (e.g. MFI cutoff values used for antibody detection), availability of prospective DSA monitoring, adjustment for significant confounders (e.g. immunosuppression regimen, patient adherence) and methodological model used for determining the association between HLA immunogenicity score and DSA response (e.g. immunogenicity score of individual HLA mismatches and DSA response to the same antigen vs. total immunogenicity score at an HLA locus level and alloantibody response to any of the locus mismatches vs. total immunogenicity score of all donor–recipient HLA mismatches and alloantibody response to any of these mismatches). International collaboration and setting up of multicenter consortia will be required to address these sources of bias and facilitate the design of large, appropriately powered, datasets to investigate the predictive power of HLA immunogenicity algorithms and how they might be applied in the clinical setting. Future studies should focus on investigating how HLA immunogenicity algorithms might be applied to improve organ allocation policies (provided that a clear benefit over conventional HLA matching at the antigen level is demonstrated) and model how any changes might impact equity of access to transplantation. In addition, prospective studies will be needed to evaluate the potential of HLA immunogenicity analysis to assess immunological risk and facilitate clinical decision making at individual patient level [48]. There is also an impetus on continuing improvement of currently available algorithms and/or development of new approaches. This relates to both B-cell and T-cell HLA immunogenicity algorithms which might be further improved by better definition of the structure and physicochemical properties of HLA B-cell epitopes (e.g. through experimental resolution of alloantibody-HLA structures and computational approaches [49]), more accurate prediction of T-cell epitopes (including experimental epitope validation and cross-fertilization from relevant fields [50–52]), and from development of combined approaches that incorporate all aspects of alloreactivity.

## Acknowledgements

None.

### Financial support and sponsorship

The current study was supported by the Cambridge NIHR Biomedical Research Centre and the NIHR Blood and Transplant Research Unit in Organ Donation and Transplantation at the University of Cambridge in collaboration with Newcastle University and in partnership with NHS Blood and Transplant (NHSBT). The views expressed are those of the authors and not necessarily those of the NHS, the NIHR, the Department of Health or NHSBT. V.K. was supported by an Evelyn Trust Grant and an NIHR Post-Doctoral Fellowship (PDF-2016-09-065).

### Conflicts of interest

There are no conflicts of interest.

## REFERENCES AND RECOMMENDED READING

Papers of particular interest, published within the annual period of review, have been highlighted as:

▪ of special interest
▪▪ of outstanding interest
